# Pretreatment with AM1241 Enhances the Analgesic Effect of Intrathecally Administrated Mesenchymal Stem Cells

**DOI:** 10.1155/2019/7025473

**Published:** 2019-09-10

**Authors:** Junran Xie, Jinxuan Ren, Na Liu, Chengwei Wu, Dongju Xiao, Huanyu Luo, Jingxian Du

**Affiliations:** ^1^Department of Anesthesiology, Sir Run Run Shaw Hospital, School of Medicine, Zhejiang University, Hangzhou 310020, China; ^2^Department of Anesthesiology, Second Affiliated Hospital, School of Medicine, Zhejiang University, Hangzhou 310016, China; ^3^Department of Anesthesiology, Li Shui Hospital of Zhejiang University, Lishui 323000, China; ^4^Jiangsu Province Key Laboratory of Anesthesiology, Xuzhou Medical University, Xuzhou 221004, China

## Abstract

Mesenchymal stem cells have cannabinoid (CB) receptors type 1 and type 2 and can alleviate a variety of neuropathic pains, including chronic constriction injury (CCI). A selective CB2 receptor agonist is AM1241. In the present study, it was found that mice with CCI displayed a longer duration of mechanical and thermal analgesia when intrathecally (i.t.) injected with AM1241-treated mesenchymal stem cells, compared to those injected with untreated mesenchymal stem cells or AM1241 alone. Moreover, CCI-induced upregulation of the phosphorylated extracellular signal-regulated kinase (ERK) 1/2 (p-ERK1/2) was inhibited following i.t. injection of AM1241-treated mesenchymal stem cells and this inhibition was noticeably higher compared to injection with untreated mesenchymal stem cells. The expression of transforming growth factor-*β*1 (TGF-*β*1) was also analyzed in the dorsal root ganglion (DRGs) and spinal cord of CCI mice. In untreated CCI mice, expression of TGF-*β*1 was increased, whereas pretreatment with AM1241-treated mesenchymal stem cells regulated the expression of TGF-*β*1 on 10 days and 19 days after surgery. In addition, i.t. injection of exogenous TGF-*β*1 slightly alleviated neuropathic pain whilst neutralization of TGF-*β*1 potently blocked the effect of AM1241-treated mesenchymal stem cells on thermal hyperalgesia and mechanical allodynia of CCI mice. In an in vitro experiment, AM1241 could enhance the release of TGF-*β*1 in the supernatant of BMSCs after lipopolysaccharide (LPS) simulation. Taken together, the findings of the current study show that i.t. administration of AM1241-treated mesenchymal stem cells has a positive effect on analgesia and that TGF-*β*1 and p-ERK1/2 may be the molecular signaling pathway involved in this process.

## 1. Introduction

Neuropathic pain is chronic pain caused by nervous system damage or dysfunction, including chronic constriction injury (CCI), trauma, infection, tumor (bone cancer), metabolic disease (diabetes), chemotherapy drugs (paclitaxel for example) [1–3], the peripheral sensory nerve (peripheral sensitization), and changes in the central sensory nerves (central sensitization) of the spinal cord or brain that lead to increased sensitivity to pain [4]. Neuropathic pain is a kind of intractable pain, as even if the initial damaged tissue is cured, the pain will last for several months to several years [5]. Due to complex pathogenesis and currently ineffective treatment [3, 6], the treatment of neuropathic pain urgently needs to be investigated.

In recent years, stem cell transplantation has been regarded as an effective therapeutic method for the treatment of neuropathic pain [7]. Mesenchymal stem cells are the foremost source of cell therapy, derived from the umbilical cord [8], placenta, bone marrow [9], and adipose tissue [10], to name a few sources. With their extensive ability to differentiate and migrate, mesenchymal stem cells, once injected to the injured site, can promote tissue and nerve repair. In particular, bone marrow mesenchymal stem cells (BMSCs) easily grow in vitro and elicit immunomodulatory features and multipotentiality with high genetic stability [7, 11]. The injection of bone marrow-derived mesenchymal stem cells (BM-MSCs) to the injured spinal cord results in a significant improvement in motor function of the hind limbs and shows solid immunosuppressive, antiproliferative, anti-inflammatory, and antiapoptotic characteristics [9]. In addition, previous studies have demonstrated that cannabinoid (CB) receptors exist on the surface of bone marrow-derived mesenchymal stem cells (BM-MSCs), including cannabinoid receptors type 1 and type 2 (CB1 and CB2, respectively) [12]. The expression of CB2 in BMSCs increased their differentiation and maturation [12]. Moreover, CB2 receptors were shown to be upregulated in the central nervous system (spinal cord) and peripheral nervous system (dorsal root ganglion) after pathological pain [13–15]. Several studies have confirmed CB2 as a therapeutic approach for treating chronic pain with the absence of side effects [13–15].

It has been suggested that mesenchymal stem cells act as a the “drugstore” for injury [16], as it has been demonstrated that they release cytokines and trophic factors with therapeutic effects [17, 18]. However, many significant problems with mesenchymal stem cells being used as a potential treatment for injury remain. In particular, the definite molecular mechanisms by which BMSCs inhibit chronic pain remains unknown and improvement in the therapeutic effect of BMSCs on neuropathic pain requires further investigation.

In the present study, intrathecal (i.t.) administration of pretreated BMSCs with CB2 receptor agonist, AM1241, in a mouse model of CCI, prolonged the relief time of neuropathic pain, but not the relief effect, compared with i.t. administration of BMSCs or AM1241 alone. The expression of phosphorylated extracellular signal-regulated kinase (ERK) 1/2 (p-ERK1/2) was significantly increased in the CCI model; however, i.t. administration of BMSCs, AM1241, and AM1241-pretreated BMSCs reversed the CCI-induced p-ERK1/2, with a significantly greater effect on the AM1241-pretreated BMSC group compared with the BMSC and AM1241 groups. This study also showed that transforming growth factor-*β*1 (TGF-*β*1) can regulate p-ERK1/2 in the dorsal root ganglion (DRGs) and spinal cord. The results of this study reveal that AM1241-pretreated BMSCs can alleviate neuropathic pain, compared to BMSCs or AM1241 alone, and that p-ERK1/2 and TGF-*β*1 may be involved in this process.

## 2. Materials and Methods

### 2.1. Animals

Adult male *C57BL/6* mice (approximately 20 g) were purchased from Nanjing Institute of Biomedicine and used for behavioral studies and tissue samples. All mice were housed in a specific pathogen-free vivarium under a 12 h light-dark cycle. Mice were allowed to access to food and water ad libitum. This study was carried out in accordance with the National Institute of Health Guide for the Care and Use of Laboratory Animals. All experimental procedures were approved by the Animal Research Committee of the Second Affiliated Hospital, School of Medicine, Zhejiang University (Hangzhou, China).

### 2.2. Reagents

AM1241 was purchased from Selleck; TGF-*β*1 was from BioLegend; TGF-*β*-neutralizing antibody (Ab) was from RD; LPS was from Sigma. Mesenchymal stem cells were kindly provided by the Stem Cell Bank, Chinese Academy of Sciences. The BMSCs were cultured to the fifth generation for i.t. administration. The BMSCs were pretreated with AM1241 (0.5 *μ*M) for 24 h. The TGF-*β*1 was dissolved in phosphate-buffered saline (PBS) containing 0.01% dimethyl sulfoxide (DMSO) to a final concentration of 10 ng/mL. The TGF-*β*1 (10 ng/mL) and TGF-*β*-neutralizing Ab (4 *μ*g) were separately injected in vivo. LPS was dissolved in phosphate-buffered saline (PBS) to a final concentration of 100 ng/mL. For i.t. injections, the reagents (10 *μ*L) or cells (2-2.5 × 10^5^ cells in 10 *μ*L PBS) were injected in the L3 and L4 levels of the spinal cord via a 25-gauge needle.

### 2.3. Neuropathic Pain Model

Chronic constriction injury (CCI) was used as the neuropathic pain model [11]. Briefly, the CCI model required constriction of the left sciatic nerve in the mice. Animals were anesthetized with isoflurane, the left sciatic nerve was exposed, and 4 ligatures (6-0 prolene) were placed around the sciatic nerve with 1 mm between each ligature. The ligatures were carefully tied until a short flick of the ipsilateral hind limb was observed, and then the skin incision was sutured. For the sham-operated group, the left sciatic nerve was exposed but ligature was not performed.

### 2.4. Behavioral Testing

Animals were habituated to the testing environment for at least 2 days before baseline testing. The temperature and humidity of the room remained identical for all experiments. Thermal sensitivity was tested using the Hargreaves method using a heat radiation stimulator-irradiated mouse plantar. The time from the start of irradiation to the time when the leg was shunted in mice was considered the reflex latency caused by the heat, as previously described [19, 20]. The intensity of thermal stimulation was consistent throughout the experiment. The automatic shutoff time was 20 sec to avoid tissue damage. To avoid or reduce the effect of the previous stimulus on the subsequent stimulus effect, the same site was stimulated 3 times. The average of three consecutive measurements was regarded as the piecewise linear (PWL) value for the mouse. Mechanical sensitivity was measured with a von Frey filament. A series of normalized von Frey cilia (0.01–2.56 g) were used to vertically stimulate the middle part of the left foot of mice until they were curved into an S shape for a duration of ≤6 sec, with the appearance of raising the foot or licking their feet considered a positive reaction. The intensity of 0.16 g was used from the beginning until it no longer caused a positive reaction, and then the next higher intensity was used. If a positive reaction was observed using a small force adjacent to the level, the first positive reaction and negative reaction rides were measured 5 times. This “up-down” method was used to calculate the 50% response threshold. The average of three measurements was considered the paw withdrawal threshold (PWT) value of the mouse as previously described [21]. A rotarod test was used to assess motor function. Mice were tested 3 times by 10-minute intervals. During the tests, the speed of rotation was accelerated from 2 to 20 rpm during the test, in a total time of 5 minutes. The fall latency was recorded and averaged as previously described [11].

### 2.5. Western Blot (WB)

Dorsal spinal cords from ridge cone segment (L3-5) and DRGs were harvested from mice 5 and 14 days after i.t. injection. Protein was separated on 10% Tris-Tricine SDS-PAGE gel and transferred to polyvinylidene difluoride (PVDF) membranes (Amersham Bioscience, USA). After blocking with bovine serum albumin 5%, membranes were incubated overnight at 4°C with mouse antibodies against GAPDH (1 : 1000, Proteintech, USA) and TGF-*β*1 (1 : 1000, Abcam, USA) and rabbit Ab against p-ERK 1/2 (1 : 1000, Cell Signaling Technology, USA) and ERK 1/2 (1 : 1000, Cell Signaling Technology, USA). This was followed by incubation with HRP-conjugated secondary antibodies: goat anti-mouse or goat anti-rabbit IgG (1 : 5000, Beyotime, China) for 1 h at room temperature. Blots were detected by chemiluminescence using an X-ray film. The results were measured for grey scale densitometric analysis using ImageJ as previously described [22].

### 2.6. Immunohistofluorescence Analyses

Animals were anesthetized with isoflurane and perfused slowly through the ascending aorta with 0.9% saline, followed rapidly by 4% paraformaldehyde, and the right auricle cut. After perfusion, spinal cord segments (L3-5) and DRGs were removed and post fixed in 4% paraformaldehyde and subsequently allowed to equilibrate in 30% sucrose in PBS overnight at 4°C. The spinal cord (10 *μ*m) and DRGs sections (10 *μ*m) were incubated in PBS containing 5% normal goat serum and 0.3% TritonX-100 at room temperature for 1 h. The samples were incubated overnight at 4°C with the following primary antibodies: mouse NeuN (1 : 200, Abcam, USA) and rabbit p-ERK1/2 (1 : 200, Cell Signaling Technology, USA), followed by donkey anti-rabbit and anti-mouse secondary antibodies conjugated with Alexa Fluor 594, 488 (1 : 500; Thermo Fisher Scientific, Waltham, MA, USA). The 4′,6-diamidino-2-phenylindole DAPI DNA stain (Sigma-Aldrich, USA) was used to stain cell nuclei. Images were captured using a fluorescent microscope (DMIRB, Leica, Germany). The intensity of fluorescence was analyzed using ImageJ [23].

### 2.7. Cell Culture and Conditioned Medium Collection

Bone marrow MSCs were cultured in DMEM-F12 (Sigma) supplied with 10% FBS (Gibco, Carlsbad, CA) and 1% antibiotics (Penicillin G 10000 units/mL, streptomycin 100 *μ*g/mL) in the humidified CO_2_ incubator at 37°C. After 24 h of treatment separately with AM1241 and LPS, supernatants from BMSC cultures were collected and centrifuged at 3000 rpm for 5 min to remove remaining cells. BMSCs were also collected.

### 2.8. Enzyme-Linked Immunosorbent Assay (ELISA)

The protein expression of TGF-*β*1 was determined by the corresponding ELISA kit (Nanjing Jiancheng Bioengineering Institute, China) according to the instructions. The supernatants were collected and centrifuged at 3000 rpm for 20 min to remove remaining cells again. 50 *μ*L culture medium was used. The standard curve was included in each experiment.

### 2.9. Statistical Analyses

All data were analyzed as mean ± SEM. Group differences were calculated using one-way analyses of variance (ANOVA). The differences were further investigated using least significant difference (LSD) post hoc tests. A *P* value of less than 0.05 was considered statistically significant.

## 3. Results

### 3.1. Effects of AM1241-Pretreated BMSCs via the i.t. Route on CCI-Induced Neuropathic Pain Behavior

**Figure 1:**
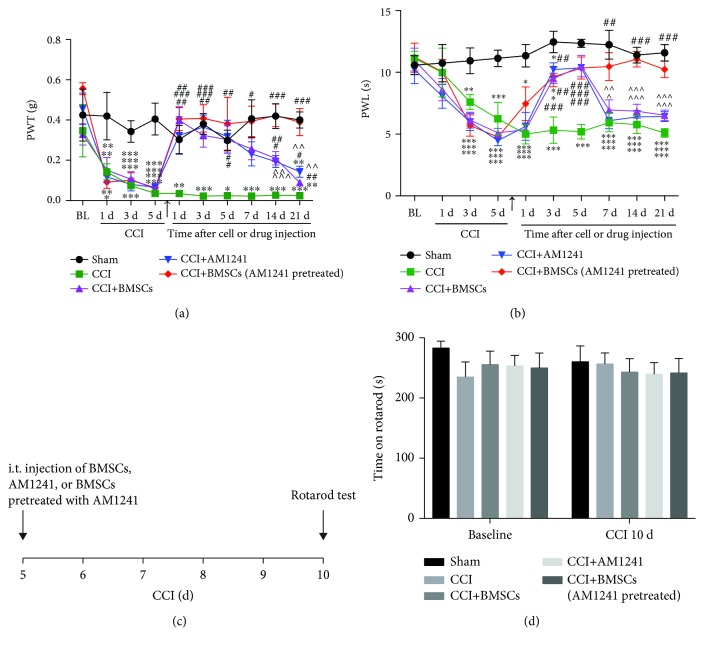
Inhibition of AM1241-pretreated BMSCs via the i.t. route on CCI-induced neuropathic pain behavior. (a) Prolonged analgesia of mechanical allodynia and (b) thermal hyperalgesia for 3 weeks after the treatment with i.t. injection of AM1241-pretreated BMSCs, given 5 days after CCI. ^∗^*p* < 0.05, ^∗∗^*p* < 0.01, and ^∗∗∗^*p* < 0.001 versus the sham group; ^#^*p* < 0.05, ^##^*p* < 0.01, and ^###^*p* < 0.001 versus the CCI group; ^∧^*p* < 0.05, ^∧∧^*p* < 0.01, and ^∧∧∧^*p* < 0.001 versus the AM1241-pretreated BMSC group; *n* = 6 mice/group. Arrows in (a) and (b) indicate the time of BMSC, AM1241, and BMSCs pretreated with AM1241 injections. (c, d) Rotarod test for the evaluation of motor function at day 10 after CCI; *n* = 6 mice/group.

Neuropathic pain was induced in mice by CCI of the left sciatic nerve. The analgesic effects of BMSCs, CB2 receptor agonist AM1241, and AM1241-pretreated BMSC were then assessed. The BMSCs (2.5 × 10^5^ cells), AM1241 (0.5 *μ*M), and BMSCs (2.5 × 10^5^ cells) pretreated with AM1241 (0.5 *μ*M) were injected via i.t. administration at day 5 after left sciatic nerve injury. Pain behavior was evaluated at 0, 1, 3, and 5 days after CCI and 1, 3, 5, 7, 14, and 21 days after injection. Administration of AM1241-pretreated BMSCs in the CCI mice inhibited thermal hyperalgesia, which lasted 3 weeks; however, the antinociceptive effects of BMSCs or AM1241 alone on the CCI mice lasted just a few days with no obvious difference between these two groups. Both the AM1241-pretreated BMSCs and AM1241 alone reduced mechanical allodynia until day 21, with AM1241 having a lower antinociceptive compared with AM1241-pretreated BMSCs. In contrast, BMSCs alone reduced mechanical allodynia until only day 14 (Figures 1(a) and 1(b)). Interestingly, i.t. injections of BMSCs, AM1241, and AM1241-pretreated MSCs did not affect motor function at day 10 after CCI in a rotarod test (Figures 1(c) and 1(d)). Collectively, these data suggest that i.t. injection of AM1241-pretreated BMSCs resulted in a longer relief of neuropathic pain than i.t. injection of BMSCs or AM1241 alone, without influencing motor function.

### 3.2. Expression of p-ERK1/2 in the DRGs and Spinal Dorsal Horn after i.t. Injections

**Figure 2:**
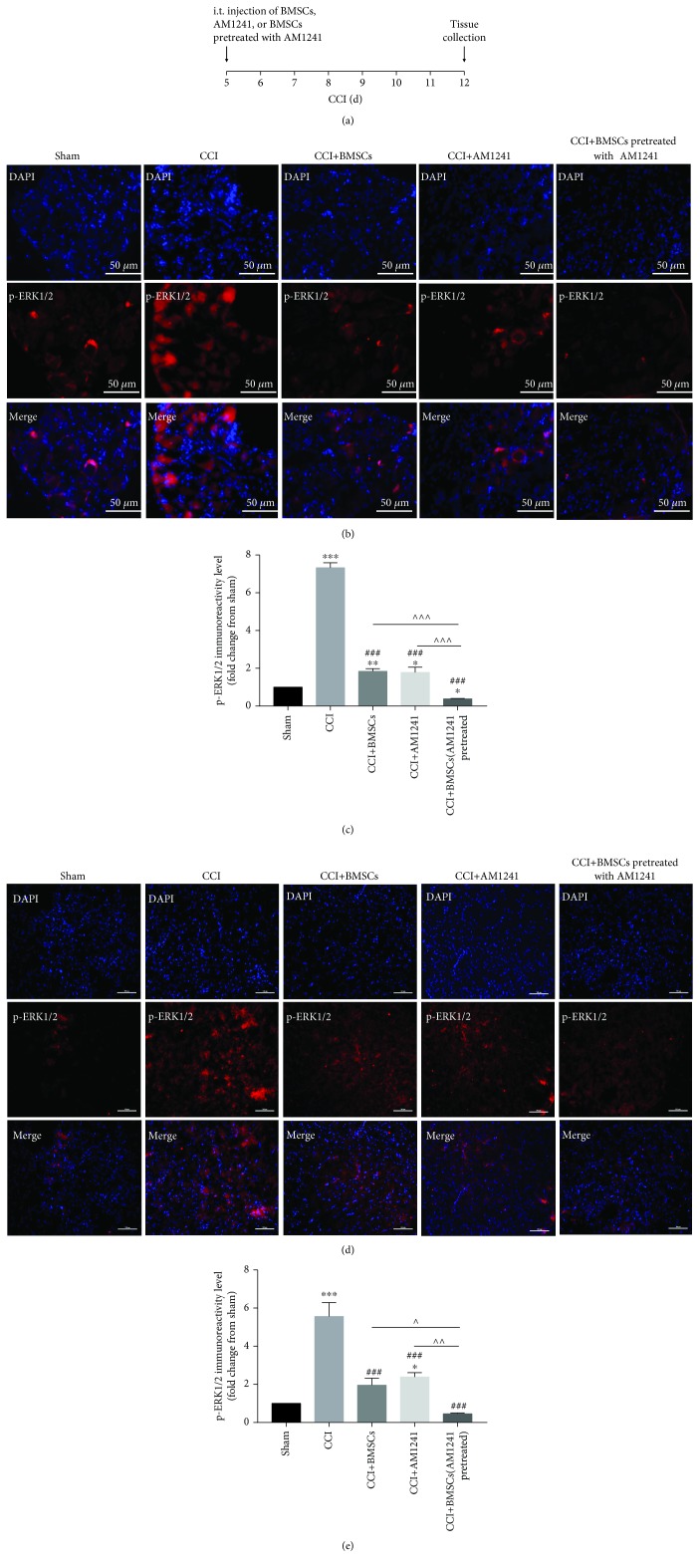
Inhibition of p-ERK1/2 in the DRGs and spinal cord dorsal horn after treatment. (a) Timeline indicating the time of BMSC, AM1241, and BMSCs pretreated with AM1241 (CCI day 5) and tissue collection (CCI day 12). (b–e) Inhibition of CCI-induced upregulation of p-ERK1/2 by i.t. injection of BMSCs pretreated with AM1241 (5 days after CCI) in the DRGs (b, c) and in the spinal dorsal horn (d, e) at CCI day 12. Quantification of p-ERK1/2 staining in the DRGs (c) and in the spinal dorsal horn (e). Scale bar for (b), 50 *μ*m; scale bar for (d), 100 *μ*m. ^∗^*p* < 0.05, ^∗∗^*p* < 0.01, and ^∗∗∗^*p* < 0.001 versus the sham group; ^#^*p* < 0.05, ^##^*p* < 0.01, and ^###^*p* < 0.001 versus the CCI group; ^∧^*p* < 0.05, ^∧∧^*p* < 0.01, and ^∧∧∧^*p* < 0.001 versus the BMSCs pretreated with AM1241 group; *n* = 4 mice/group.

It is well recognized that the mitogen-activated protein kinase (MAPK) pathway is strongly implicated in the genesis of neuropathic pain. A subgroup of the MAPK, ERK1/2, plays a crucial role in the induction and maintenance of chronic pain [24–27]. It was hypothesized in the current study that ERK1/2 may play an important role in this pain transmission process. To investigate this hypothesis, the expression level of p-ERK1/2 in the DRGs and spinal dorsal horn was investigated 7 days after injection in the sham mice, CCI, CCI+BMSCs, CCI+AM1241, and CCI+AM1241-pretreated BMSC groups (Figures 2(a)–2(e)). Treatment with i.t. BMSCs (2.5 × 10^5^ cells), AM1241 (0.5 *μ*M), and BMSCs (2.5 × 10^5^ cells) pretreated with AM1241 (0.5 *μ*M), given at day 5 after induction of CCI, inhibited CCI-induced p-ERK1/2 expression in the DRGs 12 days after CCI; however, treatment with BMSCs pretreated with AM1241 significantly inhibited p-ERK1/2 compared to i.t. BMSCs or AM1241 and the sham group (Figures 2(b) and 2(c)). Similar results were observed in the spinal dorsal horn, where treatment with BMSCs pretreated with AM1241 significantly inhibited p-ERK1/2 compared to i.t. BMSCs or AM1241 group (Figures 2(d) and 2(e)).

### 3.3. The Role of TGF-*β*1 as an Antinociceptive Factor in Neuropathic Pain

**Figure 3:**
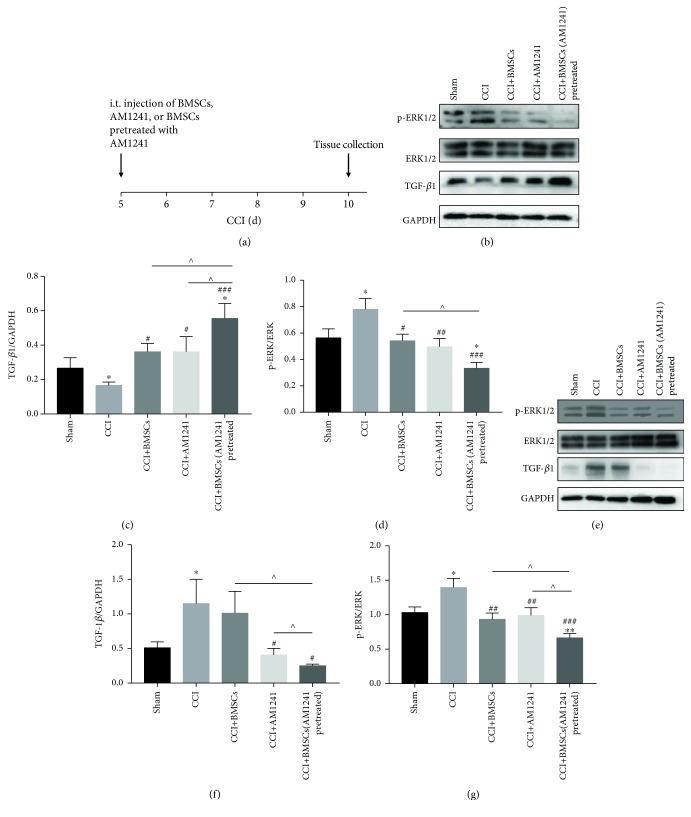
TGF-*β*1 and p-ERK1/2 expression during the early treatment. (a) Timeline shows the time of BMSC, AM1241, and BMSCs pretreated with AM1241 treatment (CCI day 5) and tissue collection (CCI day 10). (b–g) WB analysis of TGF-*β*1, p-ERK1/2, ERK1/2, and GAPDH expression after BMSC, AM1241, and BMSCs pretreated with AM1241 injections in the DRGs (b–d) and spinal cord (e–g) in the early phase of treatment. GAPDH was used as the control. ^∗^*p* < 0.05, ^∗∗^*p* < 0.01, and ^∗∗∗^*p* < 0.001 versus the sham group; ^#^*p* < 0.05, ^##^*p* < 0.01, and ^###^*p* < 0.001 versus the CCI group; ^∧^*p* < 0.05, ^∧∧^*p* < 0.01, and ^∧∧∧^*p* < 0.001 versus the BMSCs pretreated with AM1241 group; *n* = 6 mice/group.

**Figure 4:**
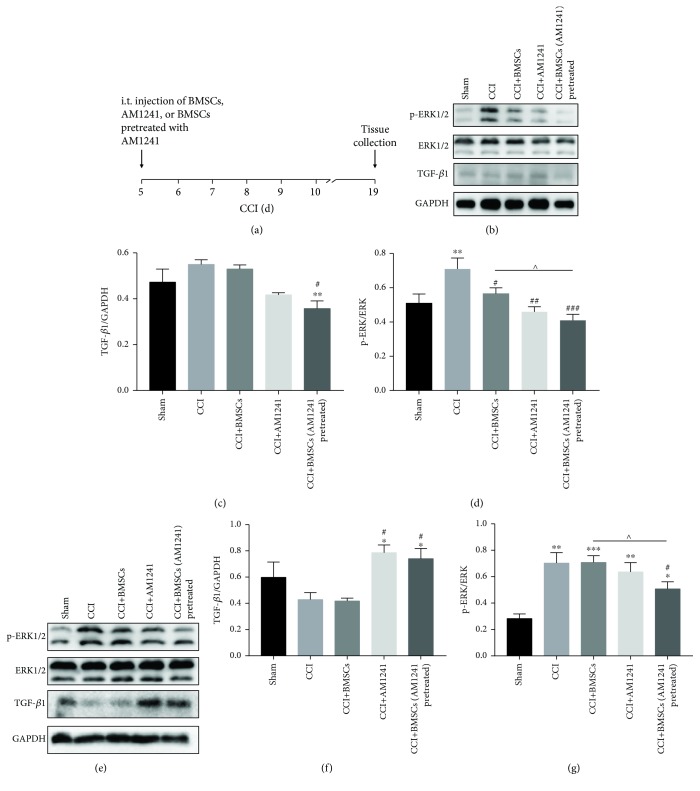
TGF-*β*1 and p-ERK1/2 expression during the late treatment. (a) Timeline shows the time of BMSC, AM1241, and BMSCs pretreated with AM1241 treatment (CCI day 5) and tissue collection (CCI day 19). (b–g) WB analysis of TGF-*β*1, p-ERK1/2, ERK1/2, and GAPDH expression after BMSC, AM1241, and BMSCs pretreated with AM1241 injections in the DRGs (b–d) and spinal cord (e–g) in the late phase of treatment. GAPDH was used as the control. ^∗^*p* < 0.05, ^∗∗^*p* < 0.01, and ^∗∗∗^*p* < 0.001 versus the sham group; ^#^*p* < 0.05, ^##^*p* < 0.01, and ^###^*p* < 0.001 versus the CCI group; ^∧^*p* < 0.05, ^∧∧^*p* < 0.01, and ^∧∧∧^*p* < 0.001 versus the BMSCs pretreated with AM1241 group; *n* = 6 mice/group.

Previous studies have shown that BMSCs can secrete several antinociceptive facts [28] including, but not limited to, hepatocyte growth factor (HGF), vascular endothelial growth factor (VEGF), chemerin, angiopoietin-1 (Ang-1), and TGF-*β*1. The cytokine, TGF-*β*1, has been implicated in the effectiveness of BMSC treatment in inflammatory disease, neuropathic pain, and immunomodulation [11, 29, 30]. In particular, in recent years, it has been reported that anti-inflammatory TGF-*β*1 may exert antinociceptive effects of its own accord. Consequently, the relationship between the expression of TGF-*β*1 and the expression of p-ERK1/2 protein was investigated. The amount of protein of TGF-*β*1 and p-ERK1/2 in the DRGs and spinal cord was measured via Western blot at days 5 and 14 after injection in the sham, CCI, CCI+BMSC, CCI+AM1241, and CCI+BMSCs pretreated with AM1241 groups (Figures 3(a)–3(g) and 4(a)–4(g)). At day 5 after injection, i.t. injection of BMSCs pretreated with AM1241, BMSCs, or AM1241 decreased the amount of p-ERK1/2 protein in the DRGs and spinal cord; however, the inhibitory effect of treatment with BMSCs pretreated with AM1241 was significant compared with that observed in the BMSC group in the DRGs and both the BMSC and AM1241 groups in the spinal cord (Figures 3(b) and 3(d) for DRGs and Figures 3(e) and 3(g) for the spinal cord). At day 5 after injection, i.t. injection of BMSCs pretreated with AM1241, BMSCs, or AM1241 increased the amount of TGF-*β*1 protein in the DRGs with the increase in protein being significant in the BMSCs pretreated with AM1241 group compared with the other two groups and the sham group (Figures 3(b) and 3(c)). In the spinal cord at day 5 after injection, there was no significant difference between the BMSC group and the CCI alone group or the sham group. The levels of TGF-*β*1 protein in the BMSCs pretreated with AM1241 group and the AM1241 group were significantly reduced compared to that of the CCI alone group, and the BMSCs pretreated with AM1241 group was significant compared with the BMSC group (Figures 3(e) and 3(f)). At day 14 after injection, the level of p-ERK1/2 protein was reduced in the BMSCs pretreated with AM1241 group compared with the CCI alone group and this was significant compared with the BMSC group in both the DRGs and the spinal cord (Figures 4(b) and 4(d) for the DRGs and Figures 4(e) and 4(g) for the spinal cord). In contrast to day 5, at day 14 in the DRGs, the level of TGF-*β*1 was significantly decreased in the BMSCs pretreated with AM1241 group (Figures 4(b) and 4(c)). At day 14 in the spinal cord, this was reversed with an increase in the level of TGF-*β*1 protein in the BMSCs pretreated with AM1241 group (Figures 4(e) and 4(f)).

### 3.4. Exogenous TGF-*β*1 Potently Attenuates Neuropathic Pain through Inhibition of p-ERK1/2

**Figure 5:**
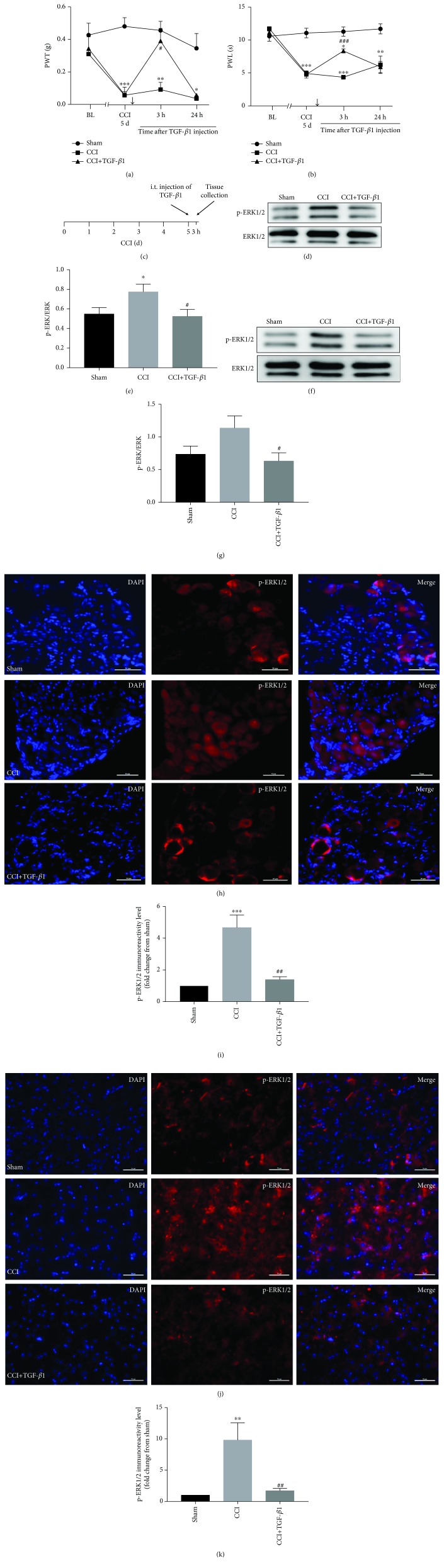
Expression of p-ERK following administration of exogenous TGF-*β*1. (a) The reversal of CCI-induced mechanical allodynia and (b) thermal hyperalgesia at 3 h after i.t. injection of exogenous TGF-*β*1. (c) Timeline shows the time of TGF-*β*1 treatment (CCI day 5) and tissue collection (3 h after injection). Arrows indicate the time of exogenous TGF-*β*1 injection. Inhibition of CCI-induced upregulation of p-ERK1/2 by i.t. injection of exogenous TGF-*β*1 (CCI day 5) via WB in the (d, e) DRGs and in the (f, g) spinal cord; *n* = 6 mice/group. (h–k) Immunohistofluorescence analysis of p-ERK1/2 after i.t. injection of exogenous TGF-*β*1 (CCI day 5) in the (h, i) DRGs and in the (j, k) spinal dorsal horn; *n* = 4 mice/group. Quantification results of p-ERK1/2 staining in the (i) DRGs and in the (k) spinal dorsal horn. Scale bar, 50 *μ*m. ^∗^*p* < 0.05, ^∗∗^*p* < 0.01, and ^∗∗∗^*p* < 0.001 versus the sham group; ^#^*p* < 0.05, ^##^*p* < 0.01, and ^###^*p* < 0.001 versus the CCI group.

To further investigate the hypothesis that TGF-*β*1 plays a role in the reduction in the level of p-ERK1/2, thus alleviating neuropathic pain, the treatment effects of exogenous TGF-*β*1 (10 ng) on the CCI-induced mechanical allodynia and heat hyperalgesia were explored. Pain behavior was evaluated at 0 and 5 days after CCI and 3 and 24 h after exogenous TGF-*β*1 injection. The exogenous TGF-*β*1 delivered via i.t. significantly inhibited mechanical allodynia (Figure 5(a)) and thermal hyperalgesia (Figure 5(b)) at day 5 after CCI. This reversal remained transient at the 3 h after injection, recovering after 24 h (Figures 5(a) and 5(b)).

The level of p-ERK1/2 protein was measured by Western blot and the protein expression by immunohistofluorescence in the DRGs and spinal dorsal horn at 3 h after exogenous TGF-*β*1 injection (Figures 5(c)–5(k), 5(d)–5(g) for Western blot and Figures 5(h)–5(k) for immunohistofluorescence). Treatment with exogenous TGF-*β*1 (10 ng) inhibited the CCI-induced p-ERK1/2 protein in both the DRGs and spinal dorsal horn at 3 h after exogenous TGF-*β*1 injection (Figures 5(d)–5(g)). Immunohistofluorescence showed a similar result in terms of p-ERK1/2 protein expression (Figure 5(h)–5(k)).

### 3.5. Neutralization of TGF-*β*1 Reversed the Analgesic Effect of BMSCs Pretreated with AM1241

**Figure 6:**
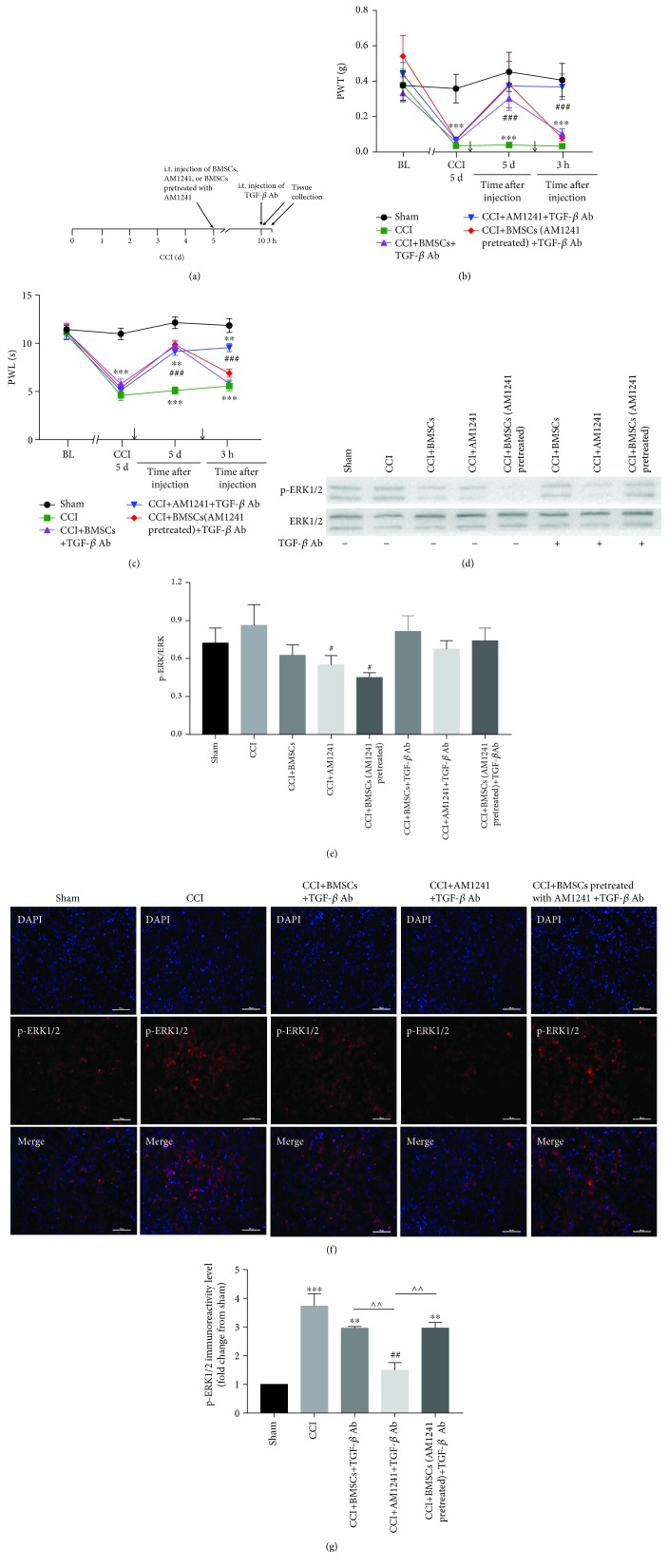
Administration of TGF-*β*1-neutralizing Ab on neuropathic pain and expression of p-ERK1/2. (a) Timeline of the time of BMSC, AM1241, and BMSCs pretreated with AM1241 injections (CCI day 5), the time of TGF-*β*-neutralizing Ab (day 5 after treatment), and tissue collection (day 5 after treatment and 3 h after TGF-*β*-neutralizing Ab injection). (b, c) The reversal of BMSC- and BMSCs pretreated with AM1241-induced (b) mechanical and (c) thermal analgesia at 3 h after i.t. injection of TGF-*β*-neutralizing Ab. (d, e) WB analysis of p-ERK1/2 expression in all treatment groups after i.t. injection of TGF-*β*-neutralizing Ab; *n* = 6 mice/group. (f, g) Expression of p-ERK1/2 via immunofluorescence in the BMSC and BMSCs pretreated with AM1241 groups in the spinal dorsal horn after i.t. injection of TGF-*β*-neutralizing Ab; *n* = 4 mice/group. (g) Quantification results of p-ERK1/2 staining. Scale bar, 100 *μ*m. ^∗^*p* < 0.05, ^∗∗^*p* < 0.01, and ^∗∗∗^*p* < 0.001 versus the sham group; ^#^*p* < 0.05, ^##^*p* < 0.01, and ^###^*p* < 0.001 versus the CCI group; ^∧^*p* < 0.05, ^∧∧^*p* < 0.01, and ^∧∧∧^*p* < 0.001 versus the AM1241+TGF-*β* Ab group. Arrows in (b) and (c) indicate the time of BMSC, AM1241, and BMSCs pretreated with AM1241 injections and the time of TGF-*β*-neutralizing Ab injection, respectively.

Mice were treated with a specific neutralizing Ab against TGF-*β* (4 *μ*g), 5 days after BMSCs, AM1241, and BMSCs pretreated with AM1241 injections (Figure 6(a)). The analgesic effects of BMSCs and BMSCs pretreated with AM1241 were reversed at 3 h after TGF-*β*-neutralizing Ab injection in mechanical allodynia and thermal hyperalgesia (Figures 6(b) and 6(c)). In contrast to the Western blot that showed an inhibition of p-ERK1/2 protein expression in the spinal cord following AM1241 and BMSCs pretreated with AM1241 injections, the TGF-*β*-neutralizing Ab blocked the analgesic effect of TGF-*β*1 and increased the expression of p-ERK1/2 protein in the BMSCs and BMSCs pretreated with AM1241; however, there was no significant difference between any of the groups (Figures 6(d) and 6(e)). In the immunohistofluorescence studies, the TGF-*β*-neutralizing Ab reversed the inhibition of p-ERK1/2 expression in the BMSC and BMSCs pretreated with AM1241 groups at 3 hours after TGF-*β*-neutralizing Ab injection (Figures 6(f) and 6(g)). The expression of p-ERK1/2 remained reduced in the AM1241 group (Figures 6(f) and 6(g)). There was no significant difference between the CCI+BMSC and CCI+AM1241-pretreated BMSC groups (Figures 6(f) and 6(g)).

### 3.6. BMSCs Pretreated with AM1241 Enhance the Secretion of TGF-*β*1 after LPS Stimulation In Vitro

**Figure 7:**
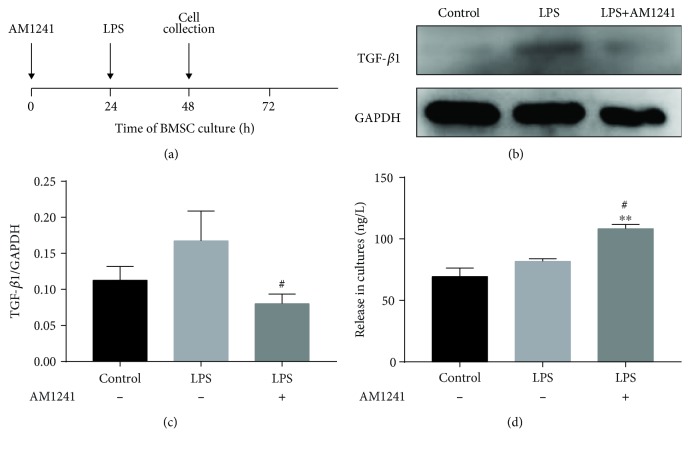
Expression of TGF-*β*1 in BMSCs pretreated with AM1241 and cell supernatant. (a) Timeline of the time of AM1241 treatment and LPS stimulation (24 h after AM1241 treatment) and cell collection (24 h after LPS stimulation). (b, c) WB analysis of TGF-*β*1 expression in three groups. (d) Release of TGF-*β*1 via ELISA in cell supernatant of three groups. ^∗^*p* < 0.05, ^∗∗^*p* < 0.01, and ^∗∗∗^*p* < 0.001 versus the control group; ^#^*p* < 0.05, ^##^*p* < 0.01, and ^###^*p* < 0.001 versus the simple LPS group.

In order to further understand the effect of BMSCs pretreated with AM1241, we verified it in an in vitro experiment. Firstly, BMSCs (1 × 10^5^ cells) were pretreated with AM1241 (0.5 *μ*m) and were stimulated by LPS (100 ng/mL) for 24 hours (Figure 7(a)). The amount of protein of TGF-*β*1 in the cells was measured via Western blot (Figures 7(b) and 7(c)). After LPS stimulation, the TGF-*β*1 expression in the pretreatment with AM1241 group decreased compared with that in the pretreatment without AM1241 group (Figure 7(c)). Then we detected the secretion level of TGF-*β*1 in the medium of BMSCs. ELISA results showed that pretreatment with AM1241 enhanced the TGF-*β*1 content from the cell supernatant of BMSCs compared to other groups (Figure 7(d)). Collectively, AM1241 could improve the TGF-*β*1 release in BMSCs under pathological conditions.

## 4. Discussion

Neuropathic pain is an increasing health concern in the world, affecting up to 30% of adults, with a large majority of common treatments for other diseases, such as chemotherapy and opioid drugs, contributing to this chronic pain. Despite the high percentage of adults suffering from this pain, current treatments remain ineffective [31]. Recently, several studies have shown that BMSCs inhibit pain in different neuropathic pain models, including but not limited to the chronic constriction injury (CCI) model, the peripheral nerve injury (PNI) model [32, 33], or the spinal cord injury (SCI) model [34]. The specific mechanisms of how BMSCs alleviate pain and how to enhance the analgesic effect of BMSCs remain unclear. In the present study, the several investigations conducted show that the analgesic effects of BMSCs are enhanced by pretreating BMSCs with the CB2 receptor agonist AM1241, which ultimately results in slight inhibition of p-ERK1/2 compared with BMSCs or AM1241 treatment alone, and that TGF-*β*1 acts as a “middle bridge” in this process by affecting the expression of p-ERK1/2.

A member of the MAPK family, ERK1/2, has been demonstrated to lead to inflammation or neuropathic pain [35, 36]. In the current study, the expression of p-ERK1/2 was significantly increased in CCI mice. It was hypothesized that the phosphorylation of ERK1/2 was suppressed after i.t. administration of BMSCs and BMSCs pretreated with AM1241 in both the DRGs and spinal dorsal horn at day 7 after injection (at day 12 after CCI) (Figure 2), with a stronger antinociceptive effect on the BMSCs pretreated with AM1241 group (Figures 1 and 2).

It was also shown in the current study that expression of the anti-inflammatory factor, TGF-*β*1, was reduced in the DRGs of CCI mice, compared with CCI mice from all treatment groups, with a significant increase in TGF-*β*1 in the BMSCs pretreated with AM1241 group at day 5 after i.t. injection (Figure 3). Interestingly, the expression of TGF-*β*1 in the spinal cord was opposite to that observed in the DRGs, indicating a different level of pain management in these two areas (Figures 3 and 4). Both central and peripheral sensitizations were then investigated [37]. When neuropathic pain was applied, the peripheral sensory neurons were the first to respond, resulting in BMSC, AM1241, and BMSCs pretreated with AM1241 treatment to modulate the secretion of TGF-*β*1, thus exerting an analgesic effect. This was followed by the pain being transmitted to the central sensory neurons, whereby TGF-*β*1 was secreted to inhibit the hypersensitivity in the spinal cord in the CCI mice and the BMSC group. Consequently, p-ERK1/2 expression was suppressed when TGF-*β*1 expression was increased in the early phase of treatment, particularly in the BMSCs pretreated with AM1241 group. In the late phase of treatment, exactly at day 14 after treatment (day 19 after CCI), p-ERK1/2 expression remained inhibited in the BMSCs pretreated with AM1241 group; however, in contrast to the early phase, TGF-*β*1 secretion in the BMSCs pretreated with AM1241 group was decreased in the late phase. This is the first report to the authors' knowledge of this contradictory phenomenon. It is hypothesized that other factors or proteins, in conjunction with TGF-*β*1, exert the analgesic effects seen in the late phase of neuropathic pain and are involved in this pathway. Along with TGF-*β*1, several members of the TGF-*β* superfamily are secreted by several cells in vivo. The molecular mechanism by which BMSCs pretreated with AM1241 enhance TGF-*β*1 may be by activating TGF-*β*1 of cells in vivo or by promoting TGF-*β*1 secretion derived from the BMSCs. Through in vitro experiment, we verified that AM1241 could promote the release of TGF-*β*1 in cell supernatant of BMSCs (Figure 7).

In an attempt to further understand the role of TGF-*β*1 in neuropathic pain, we found that the treatment of exogenous TGF-*β*1 (10 ng) attenuated neuropathic pain (Figure 5). Next, we reversed the verification. TGF-*β*-neutralizing Ab (4 *μ*g) was given (Figure 6). In the BMSC and AM1241-pretreated BMSCs groups, the treatment effect of CCI-induced neuropathic pain behavior was reversed following injection with TGF-*β*-neutralizing Ab. The expression of p-ERK1/2 was also increased at 3 h after TGF-*β* Ab injection in this two treatment groups. Several articles have shown that CB2 receptors display antinociceptive effects on different models of neuropathic pain, indicating that the CB2 receptor maybe a good candidate for clinical development of treatment for neuropathic pain [32]. *Cannabinoid*s were highly effective in suppressing pain behaviors of chemotherapy-induced neuropathic pain; however, widespread use of cannabinoids was limited due to side effects in the central nervous system (CNS) [38]. As delivery of the TGF-*β*-neutralizing Ab did not reverse the antinociceptive effects of AM1241, even though administration of AM1241 increased the secretion of TGF-*β*1, it is clear that the interaction between CB2 and TGF-*β*1 is a complicated molecular process requiring further research.

This study includes several limitations. The CCI model was employed in this investigation, and therefore, it cannot be assumed that the mechanisms observed and hypothesized in this study can be applied to other models of neuropathic pain. Therefore, it is necessary to investigate these mechanisms and possibly conduct similar experiments in other models such as the PNI model or the SCI model. All groups only received one injection, and it may be that one injection resulted in some false positive results indicating the need to investigate the outcomes of multiple injections.

## 5. Conclusions

In summary, our findings demonstrate that i.t. delivery of the BMSCs pretreated with the CB2 agonist AM2141 may enhance pain relief further than i.t. delivery of BMSCs alone by inhibiting p-ERK1/2 expression and that TGF-*β*1 may be the possible signaling molecule involved in this pathway. This strategy may be involved in the clinical development of treatment for neuropathic pain.

## Data Availability

The (original data: behavioral testing, Western blot, immunohistofluorescence, and ELISA) data used to support the findings of this study are included within the article.
